# Monoaminergic and Neuropeptidergic Neurons Have Distinct Expression Profiles of Histone Deacetylases

**DOI:** 10.1371/journal.pone.0058473

**Published:** 2013-03-04

**Authors:** Kenkichi Takase, Satoko Oda, Masaru Kuroda, Hiromasa Funato

**Affiliations:** 1 Department of Anatomy, Toho University School of Medicine, Tokyo, Japan; 2 Center for Behavioral Molecular Genetics, University of Tsukuba, Tsukuba, Japan; 3 International Institute for Integrative Sleep Medicine, University of Tsukuba, Tsukuba, Japan; Kent State University, United States of America

## Abstract

Monoaminergic and neuropeptidergic neurons regulate a wide variety of behaviors, such as feeding, sleep/wakefulness behavior, stress response, addiction, and social behavior. These neurons form neural circuits to integrate different modalities of behavioral and environmental factors, such as stress, maternal care, and feeding conditions. One possible mechanism for integrating environmental factors through the monoaminergic and neuropeptidergic neurons is through the epigenetic regulation of gene expression via altered acetylation of histones. Histone deacetylases (HDACs) play an important role in altering behavior in response to environmental factors. Despite increasing attention and the versatile roles of HDACs in a variety of brain functions and disorders, no reports have detailed the localization of the HDACs in the monoaminergic and neuropeptidergic neurons. Here, we examined the expression profile of the HDAC protein family from HDAC1 to HDAC11 in corticotropin-releasing hormone, oxytocin, vasopressin, agouti-related peptide (AgRP), pro-opiomelanocortin (POMC), orexin, histamine, dopamine, serotonin, and noradrenaline neurons. Immunoreactivities for HDAC1,-2,-3,-5,-6,-7,-9, and -11 were very similar among the monoaminergic and neuropeptidergic neurons, while the HDAC4, -8, and -10 immunoreactivities were clearly different among neuronal groups. HDAC10 expression was found in AgRP neurons, POMC neurons, dopamine neurons and noradrenaline neurons but not in other neuronal groups. HDAC8 immunoreactivity was detected in the cytoplasm of almost all histamine neurons with a pericellular pattern but not in other neuropeptidergic and monoaminergic neurons. Thus, the differential expression of HDACs in monoaminergic and neuropeptidergic neurons may be crucial for the maintenance of biological characteristics and may be altered in response to environmental factors.

## Introduction

Monoaminergic and neuropeptidergic neurons regulate a wide variety of behaviors, such as feeding, sleep/wakefulness behavior, stress response, addiction, and social behavior. Feeding behavior is mainly regulated by orexigenic neurons containing neuropeptide Y (NPY) and agouti-related peptide (AgRP) and by anorexigenic neurons containing pro-opiomelanocortin (POMC). Both of these types of neurons are localized in the hypothalamic arcuate nucleus (ARC) [1]. Orexin (also known as hypocretin), which was originally identified as an orexigenic neuropeptide, is expressed in the lateral hypothalamic area (LHA) and plays a crucial role in sleep/wakefulness behavior, reward behavior, addiction, and body weight regulation [2]–[4]. Sleep/wakefulness behavior is also regulated by histamine neurons in the tuberomammillary nucleus (TMN), serotonin neurons in the dorsal raphe (DR), and noradrenaline neurons in the locus coeruleus (LC) [2], [5]. Dopamine neurons in the ventral tegmental area (VTA), which is involved in the reward system, also alter feeding behavior, social behavior, and sleep/wakefulness [2], [3], [5], [6]. The hypothalamic paraventricular nucleus (PVN) has neurons that contain corticotropin-releasing hormone (CRH), which constitutes the hypothalamic-pituitary-adrenal axis, a neuroendocrine system that controls stress response [3]. The PVN also contains neurons that produce oxytocin and vasopressin, which are important for social behavior [7].

Environmental factors such as stress, maternal care, and feeding conditions modulate the above-mentioned behaviors [8]–[11]. For instance, stressed animals decrease their food intake compared with non-stressed animals [8]. Male rats that had experienced neonatal maternal deprivation showed a decrease in total sleep time [11], and suppressed social interaction [12]. Recently, increasing reports suggest that the epigenetic regulation of gene expression plays a crucial role in behavioral change in response to environmental factors [13], [14]. One possible mechanism for integrating environmental factors is through the epigenetic gene regulation of monoaminergic and neuropeptidergic neurons.

Acetylation of histones is a dynamic process that regulates gene expression in response to upstream cellular signaling and also regulates multiple behaviors, including addiction, depression, age-related memory impairment, and memory recall [15]–[18]. Histone deacetylases (HDACs) remove an acetyl group from the lysine on the histone in a sequence-specific manner to repress, and in some cases enhance, gene transcription. HDACs also deacetylate nuclear and cytoplasmic proteins, including p53, STAT3, and α-tubulin [19]–[22]. The HDAC family comprises the following classes: class I (HDAC1, -2, -3, and -8), class IIa (HDAC4, -5, -7, and -9), class IIb (HDAC6 and -10), and class IV (HDAC11) [20], [23], [24]. HDAC4 and -5 show a nuclear-cytoplasmic shuttling in an activity-dependent manner in neural cells [25]. Recently, HDAC4 has been localized in the dendritic shaft and spines [26], suggesting that HDACs may work outside the cell body to regulate synaptic activity and dendritic transport. Consistently, overexpression of HDAC2 results in impaired memory formation [27]. In addition to memory formation, HDACs may play important roles in feeding and metabolism [28], [29]. Increasing attention has been given to the versatile roles of HDACs in a variety of brain functions and disorders. However, only a few reports have detailed the localization of HDACs in the brain [26], [29], [30], which prompted us to examine the distribution of the HDAC family in monoaminergic and neuropeptidergic neurons.

In the present study, we examined the subcellular distribution of HDACs in the CRH, oxytocin, vasopressin, orexin, AgRP, POMC, histamine, dopamine, serotonin, and noradrenaline neurons in mice using the immunofluorescence method.

## Materials and Methods

### Animal

Male C57BL/6J mice (25–30 g, 12–16 week-old; *n* = 6) were obtained from Charles River Laboratories of Japan (Tokyo, Japan). Mice were provided food and water *ad libitum*, maintained on a 12-hour light/dark cycle (lights on, 0800–2000 h), and housed under controlled temperature (25±1°C) and humidity conditions. All procedures were approved by the Institutional Animal Care and Use Committee of Toho University (Approved protocol ID #12-52-81).

### Antibodies

The primary antibodies used in the current study are summarized in Table 1. As markers of the oxytocin and vasopressin neurons, we used antibodies for neurophysin I and copeptin, respectively. Neurophysin I is a peptide derived from an oxytocin precursor and is a marker for oxytocin neurons. Copeptin, a peptide derived from a vasopressin precursor, co-localizes with vasopressin and not oxytocin [31]. An antibody for tyrosine hydroxylase was used as a marker of dopamine and noradrenaline neurons. The secondary antibodies used for immunofluorescent visualization were Alexa 555-conjugated anti-goat IgG antibody (1∶400, A21432, Invitrogen, CA, USA), Alexa 555-conjugated anti-guinea pig IgG antibody (1∶400, A21435, Invitrogen), or Alexa 555-conjugated anti-mouse IgG antibody (1∶400, A31570, Invitrogen), and Alexa 488-conjugated anti-rabbit IgG antibody (1∶400, A21206, Invitrogen).

**Table 1:** Primary Antibodies Used.

Neuronal group marker	Antigen	Antibody characterization or immunogen	Manufacturer, species antibodywas raised in, mono- vs. polyclonal,catalog or lot number	Dilution used
CRH	Corticotropin-releasing hormone	Affinity purified antibody forN-terminus of human CRH	Santa Cruz Biotechnology, CA; goat; polyclonal; sc-21675	1∶100
Oxytocin	Neurophysin I, a peptidederived from oxytocin precursor	Affinity purified antibody forC-terminus of mouse neurophysin I	Santa Cruz Biotechnology, CA; goat; polyclonal; sc-7810	1∶50
Vasopressin	Copeptin, a peptide derivedfrom vasopressin precursor	Affinity purified antibody forC-terminus of mouse copeptin	Santa Cruz Biotechnology, CA; goat; polyclonal; sc-7812	1∶50
Orexin	Orexin A	Affinity purified antibody for C-terminus of human orexin A	Santa Cruz Biotechnology, CA; goat; polyclonal; sc-8070	1∶100
AgRP	AgRP	Affinity purified antibody for internal region of AgRP	Santa Cruz Biotechnology, CA; goat; polyclonal; sc-18634	1∶50
POMC	POMC	Synthetic peptide: NAIIKNAYKKGE, corresponding toC terminal amino acids 256–267 of human POMC, conjugated to KLH	Abcam, Cambridge, MA; goat; polyclonal; ab32893	1∶75
Histamine	Histidine decarboxylase	Synthetic peptide corresponding to C-terminus of mouse histidine decarboxylase	American Research Products, MA; guinea pig; polyclonal; 03-16046	1∶60
Dopamine and Noradrenaline	Tyrosine hydroxylase	Tyrosine hydroxylase purified from PC12 cells	Millipore, MA; mouse; polyclonal; MAB318	1∶200
Serotonin	Serotonin	Serotonin coupled to bovine serum albumin	ImmunoStar, WI; goat; polyclonal; 20079	1∶300
	HDAC1	Synthetic peptide: KEEKPEAKGVKEEVKLA, corresponding to amino acids 466–482 of human HDAC1, conjugated to KLH	Abcam, Cambridge, MA; rabbit; polyclonal; ab19845	1∶100
	HDAC2	Synthetic peptide: GEKTDTKGTKSEQLSNP, corresponding to amino acids 471–488 of human HDAC2, conjugated to KLH	Abcam, Cambridge, MA; rabbit; polyclonal; ab16032	1∶250
	HDAC3	Synthetic peptide: NEFYDGDHDNDKESDVEI, corresponding to amino acids 411–428 of human HDAC3, conjugated to KLH	Abcam, Cambridge, MA; rabbit; polyclonal; ab16047	1∶100
	HDAC4	Synthetic peptide: CISSDPRYWYGKTQHS, corresponding to amino acids 194–209 of human HDAC4, conjugated to KLH	Abcam, Cambridge, MA; rabbit; polyclonal; ab79521	1∶300
	HDAC5	Synthetic non-phosphopeptide derived from human HDAC5 around serine 259	Abcam, Cambridge, MA; rabbit; polyclonal; ab55403	1∶100
	HDAC6	Synthetic peptide: KNIAHQNKFGEDMPH, corresponding to amino acids 1199–1213 of human HDAC6, conjugated to KLH	Abcam, Cambridge, MA; rabbit; polyclonal; ab12173	1∶50
	HDAC7	Synthetic peptide: KPRLRQIPSAEDLETDG, corresponding to amino acids 436–452 of human HDAC7 isoform a, conjugated to KLH	Abcam, Cambridge, MA; rabbit; polyclonal; ab12174	1∶50
	HDAC8	Synthetic non-phosphopeptide derived from human HDAC8, around serine 39	Abcam, Cambridge, MA; rabbit; polyclonal; ab39664	1∶250
	HDAC9	Synthetic peptide: EVPVGLEPISPLDLRT, corresponding to 55–70 of human HDAC9, conjugated to KLH	Abcam, Cambridge, MA; rabbit; polyclonal; ab18970	1∶50
	HDAC10	Synthetic peptide corresponding to a part of human HDAC10	Abcam, Cambridge, MA, Japan; rabbit; polyclonal; ab53096	1∶50
	HDAC11	Synthetic peptide, corresponding to amino acids around glycine 269 of human HDAC 11	Abcam, Cambridge, MA, Japan; rabbit; polyclonal; ab18973	1∶100
	MAP2	Bovine brain microtubule protein	Millipore, MA; mouse; polyclonal; MAB3418	1∶400
	PSD-95	Recombinant rat PSD-95	Millipore, MA; mouse; polyclonal; MAB1598	1∶400

### Immunofluorescent Staining

For immunofluorescent staining, mice (*n* = 3) were deeply anesthetized with sodium pentobarbital and perfused transcardially with phosphate-buffered saline (PBS, 0.1 M, pH 7.4) followed by phosphate-buffered 4% paraformaldehyde (PFA). Brains were rapidly removed, post-fixed overnight in phosphate-buffered 4% PFA, and equilibrated in 30% sucrose for 2 days. Brains were sectioned on a cryostat at 30 µm. Sections were stored in a cryoprotective tissue collection solution (25% glycerol, 30% ethylene glycol, 0.05 M phosphate buffer (PB)) at −20°C until use. Immunofluorescence was performed using a free-floating method. The brain sections were washed 2×10 minutes in PBS and blocked for 1 hour in a blocking solution containing 0.1 M PB, 0.25% Triton X-100, and 5% normal donkey serum. The brain sections were then incubated with primary antibodies for CRH, orexin, neurophysin I, copeptin, AgRP, POMC, histamine, tyrosine hydroxylase, serotonin, MAP-2, or PSD-95 and with antibodies for HDAC1-11 with 0.25% Triton X-100 and 3% normal donkey serum overnight at 4°C.

After washing the sections 2×20 minutes in PBS, the sections were incubated with a fluorescence-conjugated secondary antibody, and Hoechst 33342 (2 µg/ml, H21492, Invitrogen) for 2 hours at room temperature. After washing the sections 2×10 minutes in PBS and 3 minutes in PB, the sections were mounted on a glass slide with Gel/Mount (BioMeda, CA, USA). For immunofluorescent staining of CRH and AgRP neurons, colchicine was intracerebroventricularly injected into the mice (*n* = 3) one day before decapitation. Colchicine inhibits axonal transport by suppressing microtubule polymerization but does not affect nuclear-cytoplasmic shuttling.

### Confocal Laser Scanning Microscopy

Samples were observed using confocal laser scanning microscopy as previously described [32]. In brief, immunofluorescent images were captured using a scanning confocal microscope (LSM510 META, Zeiss, Oberkochen, Germany) with objectives of 40×, 63×, or 100×. The pinhole size was adjusted, so the optical thickness of the sections was 0.6–1.0 µm. To obtain immunofluorescent images, each channel was collected separately with single wavelength excitation and then merged to produce a composite image. Experimental controls were prepared in which one or both of the primary antibodies were omitted from the reaction solution. Confocal laser scanning microscopy showed no immunolabeling of omitted antibodies in the control sections. Photoshop CS5 (Adobe Systems, Mountain View, CA) was used to combine drawings and digital images into plates. The contrast and brightness of images were adjusted. HDAC immunoreactivities localized in dendrites were carefully confirmed using z-stack images.

### Image Analysis

To assess the double immunofluorescence data, we observed all CRH, oxytocin, and vasopressin neurons of the bilateral PVN of three brain sections (0.7–0.9 mm posterior to bregma; corresponding to Figures 37, 38 in Franklin and Paxinos [33]); all orexin neurons of the bilateral LHA, and all AgRP and POMC neurons of the bilateral ARC of three brain sections (1.5–1.8 mm posterior to bregma; corresponding to Figures 44–46 in Franklin and Paxinos [33]); all histamine neurons of the bilateral TMN of three brain sections (2.5–2.8 mm posterior to bregma; corresponding to Figures 52–54 in Franklin and Paxinos [33]); all dopamine neurons of the bilateral VTA of three brain sections (3.0–3.2 mm posterior to bregma; corresponding to Figures 56, 57 in Franklin and Paxinos [33]), all serotonin neurons of the DR of three brain sections (4.5–4.7 mm posterior to bregma; corresponding to Figures 68–70 in Franklin and Paxinos [33]); and all noradrenaline neurons of the bilateral LC of three brain sections (5.4–5.6 mm posterior to bregma; corresponding to Figures 76–78 in Franklin and Paxinos [33]) per animal. The percentage of HDAC-immunoreactive cells among the monoaminergic and neuropeptidergic neuron groups was determined. To verify the localization of HDACs at the subcellular level, all brain sections were counterstained with the nuclear dye Hoechst 33342. Nuclear or cytoplasmic localization of the HDACs was determined based on the colocalization with Hoechst 33342. To assess the extent of the HDACs immunoreactivities, we used a four-point scale based on the intensity and area of the immunoreactivities in the following manner: +++, strong HDACs immunoreactivity; ++, moderate HDACs immunoreactivity; +, weak HDACs immunoreactivity; and −, no HDACs immunoreactivity above background. The assessment was independently performed by two observers.

## Results

### HDAC1

**Figure 1:**
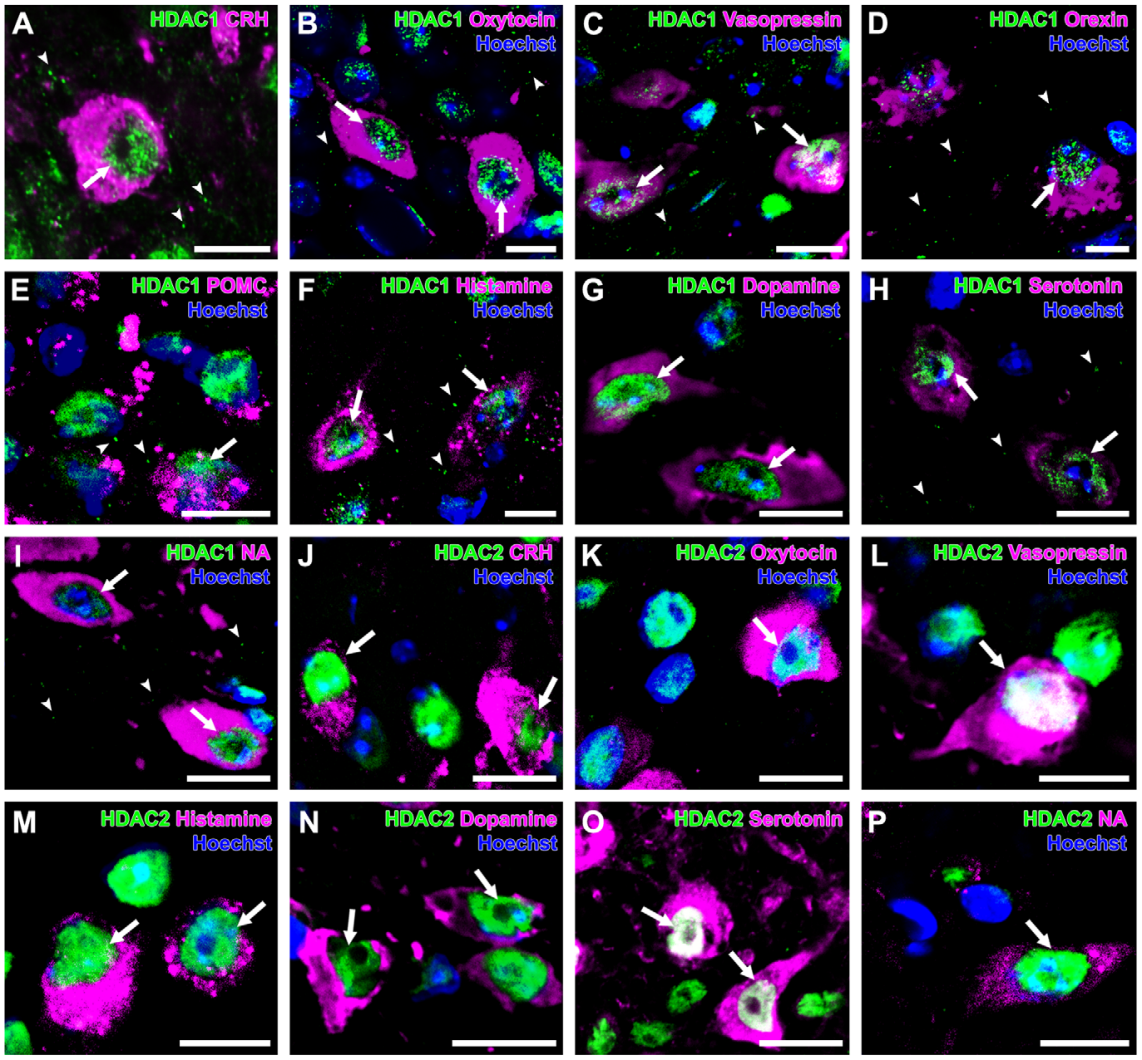
Expression of HDAC1 and -2 in monoaminergic and neuropeptidergic neurons. The HDAC immunolocalizations (green) were visualized with a neuronal marker (magenta) and Hoechst stain (blue). **A:** HDAC1 was detected in the nucleus (arrow) of a CRH neuron, with punctate immunoreactivity (arrowheads) in the PVN. **B:** HDAC1 was detected in the nuclei (arrows) of oxytocin neurons, with punctate immunoreactivity (arrowheads) in the PVN. **C:** HDAC1 was detected in the nuclei (arrows) of vasopressin neurons, with punctate immunoreactivity (arrowheads) in the PVN. **D:** HDAC1 was detected in the nucleus (arrow) of orexin neuron, with punctate immunoreactivity (arrowheads) in the LHA. **E:** HDAC1 was detected in the nucleus (arrow) of POMC neurons, with punctate immunoreactivity (arrowheads) in the ARC. **F:** HDAC1 was detected in the nuclei (arrows) of histamine neurons, with punctate immunoreactivity (arrowheads) in the TMN. **G:** HDAC1 was detected in the nuclei (arrows) of dopamine neurons. **H:** HDAC1 was detected in the nuclei (arrows) of serotonin neurons, with punctate immunoreactivity (arrowheads) in the DR. **I:** HDAC1 was detected in the nuclei (arrows) of noradrenaline neurons, with punctate immunoreactivity (arrowheads) in the LC. **J:** HDAC2 was detected in the nuclei (arrows) of CRH neurons. **K:** HDAC2 was detected in the nucleus (arrow) of oxytocin neuron. **L:** HDAC2 was detected in the nucleus (arrow) of vasopressin neuron. **M:** HDAC2 was detected in the nuclei (arrows) of histamine neurons. **N:** HDAC2 was detected in the nuclei (arrows) of dopamine neurons. **O:** HDAC2 was localized in the nuclei (arrows) of serotonin neurons in the DR. **P:** HDAC2 was detected in the nucleus (arrow) of noradrenaline neuron. Scale bars indicate 10 µm.

In general, HDAC1 immunoreactivity was recognized mainly in the nuclei of neurons and glial cells (data not shown), with punctate immunoreactivity in neuropils throughout the brain. A few HDAC1-immunoreactive puncta were also observed in the cytoplasm and dendrites. Nuclear HDAC1 immunoreactivity was found in almost all nuclei of the CRH neurons (92±6%), oxytocin neurons (95±1%), and vasopressin neurons (100±0%) of the PVN; the orexin neurons (100±0%) of the LHA; the AgRP neurons (100±0%) and POMC neurons (100±0%) of the ARC; the histamine neurons (91±6%) of the TMN; the dopamine neurons (100±0%) of the VTA; the serotonin neurons (100±0%) of the DR; and the noradrenaline neurons (100±0%) of the LC (Figures 1A–I, Table 2). HDAC1-immunoreactive puncta were uniform in size and widely distributed in the PVN, LHA, ARC, TMN, DR and LC. HDAC1-immunoreactive puncta in the neuropils were often found in dendrites and only a few HDAC1-immunoreactive puncta were colocalized with PSD95-immunoreactive puncta, which correspond to the postsynaptic area.

**Table 2:** HDACs Immunoreactivities in Monoaminergic and Neuropeptidergic Neurons.

	CRH (PVN)				Oxytocin (PVN)		
	Immunoreactive cell (%)	Subcellular immunoreactivity			Immunoreactive cell (%)	Subcellular immunoreactivity	
HDAC1	92±6	Nucleus	++	HDAC1	95±1	Nucleus	++
		Cytoplasm	+/−			Cytoplasm	+/−
HDAC2	100±0	Nucleus	+++	HDAC2	98±1	Nucleus	+++
		Cytoplasm	−∼+/−			Cytoplasm	−∼+/−
HDAC3	100±0	Nucleus	++	HDAC3	95±5	Nucleus	++
		Cytoplasm	+			Cytoplasm	+
HDAC4	100±0	Nucleus	+++	HDAC4	98±2	Nucleus	+++
		Cytoplasm	+			Cytoplasm	−∼+/−
HDAC5	100±0	Nucleus	+	HDAC5	95±5	Nucleus	+
		Cytoplasm	++			Cytoplasm	++
HDAC6	95±2	Nucleus	+++	HDAC6	70±7	Nucleus	++
		Cytoplasm	++			Cytoplasm	+
HDAC7	97±2	Nucleus	++	HDAC7	93±3	Nucleus	+
		Cytoplasm	+/−			Cytoplasm	+/−
HDAC8	0±0	Nucleus	–	HDAC8	4±3	Nucleus	–
		Cytoplasm	–			Cytoplasm	+/−
HDAC9	100±0	Nucleus	+++	HDAC9	100±0	Nucleus	+++
		Cytoplasm	+/−∼+			Cytoplasm	+
HDAC10	0±0	Nucleus	–	HDAC10	0±0	Nucleus	–
		Cytoplasm	–			Cytoplasm	–
HDAC11	45±10	Nucleus	++	HDAC11	89±8	Nucleus	++
		Cytoplasm	++			Cytoplasm	++
	**Vasopressin (PVN)**				**Orexin (LHA)**		
	**Immunoreactive cell (%)**	**Subcellular immunoreactivity**			**Immunoreactive cell (%)**	**Subcellular immunoreactivity**	
HDAC1	100±0	Nucleus	++	HDAC1	100±0	Nucleus	++
		Cytoplasm	+/−			Cytoplasm	+/−
HDAC2	100±0	Nucleus	+++	HDAC2	100±0	Nucleus	+++
		Cytoplasm	−∼+/−			Cytoplasm	−∼+/−
HDAC3	100±0	Nucleus	++	HDAC3	100±0	Nucleus	++
		Cytoplasm	+			Cytoplasm	+
HDAC4	100±0	Nucleus	+++	HDAC4	100±0	Nucleus	+++
		Cytoplasm	−∼+/−			Cytoplasm	+
HDAC5	95±3	Nucleus	+	HDAC5	100±0	Nucleus	+
		Cytoplasm	++			Cytoplasm	+++
HDAC6	80±3	Nucleus	+++	HDAC6	100±0	Nucleus	+++
		Cytoplasm	++			Cytoplasm	++
HDAC7	87±7	Nucleus	+++	HDAC7	100±0	Nucleus	+++
		Cytoplasm	+/−			Cytoplasm	+/−
HDAC8	0±0	Nucleus	–	HDAC8	0±0	Nucleus	–
		Cytoplasm	–			Cytoplasm	–
HDAC9	100±0	Nucleus	+++	HDAC9	100±0	Nucleus	+++
		Cytoplasm	+			Cytoplasm	+
HDAC10	0±0	Nucleus	–	HDAC10	0±0	Nucleus	–
		Cytoplasm	–			Cytoplasm	–
HDAC11	100±0	Nucleus	++	HDAC11	100±0	Nucleus	++
		Cytoplasm	++			Cytoplasm	++
	**AgRP (ARC)**				**POMC (ARC)**		
	**Immunoreactive cell (%)**	**Subcellular immunoreactivity**			**Immunoreactive cell (%)**	**Subcellular immunoreactivity**	
HDAC1	100±0	Nucleus	++	HDAC1	100±0	Nucleus	++
		Cytoplasm	+/−			Cytoplasm	+/−
HDAC2	100±0	Nucleus	+++	HDAC2	95±1	Nucleus	++
		Cytoplasm	−∼+/−			Cytoplasm	−∼+/−
HDAC3	100±0	Nucleus	++	HDAC3	79±11	Nucleus	++
		Cytoplasm	+			Cytoplasm	+
HDAC4	100±0	Nucleus	+++	HDAC4	92±4	Nucleus	+++
		Cytoplasm	–			Cytoplasm	–
HDAC5	100±0	Nucleus	+	HDAC5	85±1	Nucleus	+
		Cytoplasm	+++			Cytoplasm	++
HDAC6	100±0	Nucleus	+++	HDAC6	88±1	Nucleus	+++
		Cytoplasm	+			Cytoplasm	++
HDAC7	100±0	Nucleus	++	HDAC7	99±1	Nucleus	++
		Cytoplasm	+/−			Cytoplasm	+/−
HDAC8	0±0	Nucleus	–	HDAC8	0±0	Nucleus	–
		Cytoplasm	–			Cytoplasm	–
HDAC9	100±0	Nucleus	+++	HDAC9	96±4	Nucleus	+++
		Cytoplasm	+			Cytoplasm	+
HDAC10	98±2	Nucleus	++	HDAC10	55±4	Nucleus	++
		Cytoplasm	+/−			Cytoplasm	+/−
HDAC11	100±0	Nucleus	++	HDAC11	85±5	Nucleus	++
		Cytoplasm	++			Cytoplasm	++
	**Histamine (TMN)**				**Dopamine (VTA)**		
	**Immunoreactive cell (%)**	**Subcellular immunoreactivity**			**Immunoreactive cell (%)**	**Subcellular immunoreactivity**	
HDAC1	91±6	Nucleus	++	HDAC1	100±0	Nucleus	++
		Cytoplasm	+/−			Cytoplasm	+/−
HDAC2	100±0	Nucleus	+++	HDAC2	100±0	Nucleus	+++
		Cytoplasm	−∼+/−			Cytoplasm	−∼+/−
HDAC3	92±5	Nucleus	++	HDAC3	100±0	Nucleus	++
		Cytoplasm	+			Cytoplasm	+
HDAC4	85±7	Nucleus	+++	HDAC4	100±0	Nucleus	+++
		Cytoplasm	+			Cytoplasm	–
HDAC5	100±0	Nucleus	+	HDAC5	100±0	Nucleus	+
		Cytoplasm	++			Cytoplasm	++
HDAC6	100±0	Nucleus	++	HDAC6	100±0	Nucleus	+++
		Cytoplasm	+			Cytoplasm	++
HDAC7	100±0	Nucleus	++	HDAC7	100±0	Nucleus	+++
		Cytoplasm	+/−			Cytoplasm	+/−
HDAC8	100±0	Nucleus	–	HDAC8	0±0	Nucleus	–
		Cytoplasm	++			Cytoplasm	–
HDAC9	96±2	Nucleus	+++	HDAC9	100±0	Nucleus	+++
		Cytoplasm	+			Cytoplasm	++
HDAC10	0±0	Nucleus	–	HDAC10	95±1	Nucleus	++
		Cytoplasm	–			Cytoplasm	+
HDAC11	100±0	Nucleus	++	HDAC11	100±0	Nucleus	++
		Cytoplasm	++			Cytoplasm	++
	**Serotonin (DR)**				**Noradrenaline (LC)**		
	**Immunoreactive cell (%)**	**Subcellular immunoreactivity**			**Immunoreactive cell (%)**	**Subcellular immunoreactivity**	
HDAC1	100±0	Nucleus	++	HDAC1	100±0	Nucleus	++
		Cytoplasm	+/−			Cytoplasm	+/−
HDAC2	100±0	Nucleus	+++	HDAC2	100±0	Nucleus	+++
		Cytoplasm	−∼+/−			Cytoplasm	−∼+/−
HDAC3	100±0	Nucleus	++	HDAC3	100±0	Nucleus	++
		Cytoplasm	+			Cytoplasm	+/−∼+
HDAC4	93±2	Nucleus	+++	HDAC4	100±0	Nucleus	+++
		Cytoplasm	+			Cytoplasm	+
HDAC5	100±0	Nucleus	+	HDAC5	100±0	Nucleus	+
		Cytoplasm	++			Cytoplasm	+++
HDAC6	100±0	Nucleus	+++	HDAC6	100±0	Nucleus	+++
		Cytoplasm	++			Cytoplasm	++
HDAC7	100±0	Nucleus	+++	HDAC7	100±0	Nucleus	++
		Cytoplasm	+/−			Cytoplasm	+/−
HDAC8	0±0	Nucleus	–	HDAC8	0±0	Nucleus	–
		Cytoplasm	–			Cytoplasm	–
HDAC9	100±0	Nucleus	+++	HDAC9	100±0	Nucleus	+++
		Cytoplasm	+			Cytoplasm	+
HDAC10	0±0	Nucleus	–	HDAC10	100±0	Nucleus	+++
		Cytoplasm	–			Cytoplasm	++
HDAC11	100±0	Nucleus	++	HDAC11	100±0	Nucleus	++
		Cytoplasm	++			Cytoplasm	++

The percentage of immunoreactive cells was assessed at the confocal laser scanning microscopy. Values are means ± S.E.M. Nuclear or cytoplasmic localization of HDACs was determined based on the colocalization with Hoechst 33342. The extent of HDACs immunoreactivities was determined on the intensity and area of the immunoreactivities: “+++”, strong immunoreactivity; “++”, moderate immunoreactivity; “+”, weak immunoreactivity; “−“, no immunoreactivity above background.

### HDAC2

Similar to HDAC1, strong HDAC2 immunoreactivity was mainly found in the nuclei of neurons, glial cells (data not shown). Only a few HDAC2-immunoreactive puncta were observed in the hypothalamus, VTA, DR, and LC, and there were fewer HDAC2-immunoreactive puncta than for HDAC1. HDAC2 expressions was recognized in a broad range of neuron groups, including the CRH neurons (100±0%), oxytocin neurons (98±1%), and vasopressin neurons (100±0%) of the PVN; the orexin neurons (100±0%) of the LHA; the AgRP neurons (100±0%) and POMC neurons (95±1%) of the ARC; the histamine neurons of the TMN (100±0%); the dopamine neurons (100±0%) of the VTA; the serotonin neurons (100±0%) of the DR; and the noradrenaline neurons (100±0%) of the LC (Figures 1J–P, Table 2). A small number of HDAC2- immunoreactive puncta were colocalized with PSD95 immunoreactive puncta of dendrites.

### HDAC3

**Figure 2:**
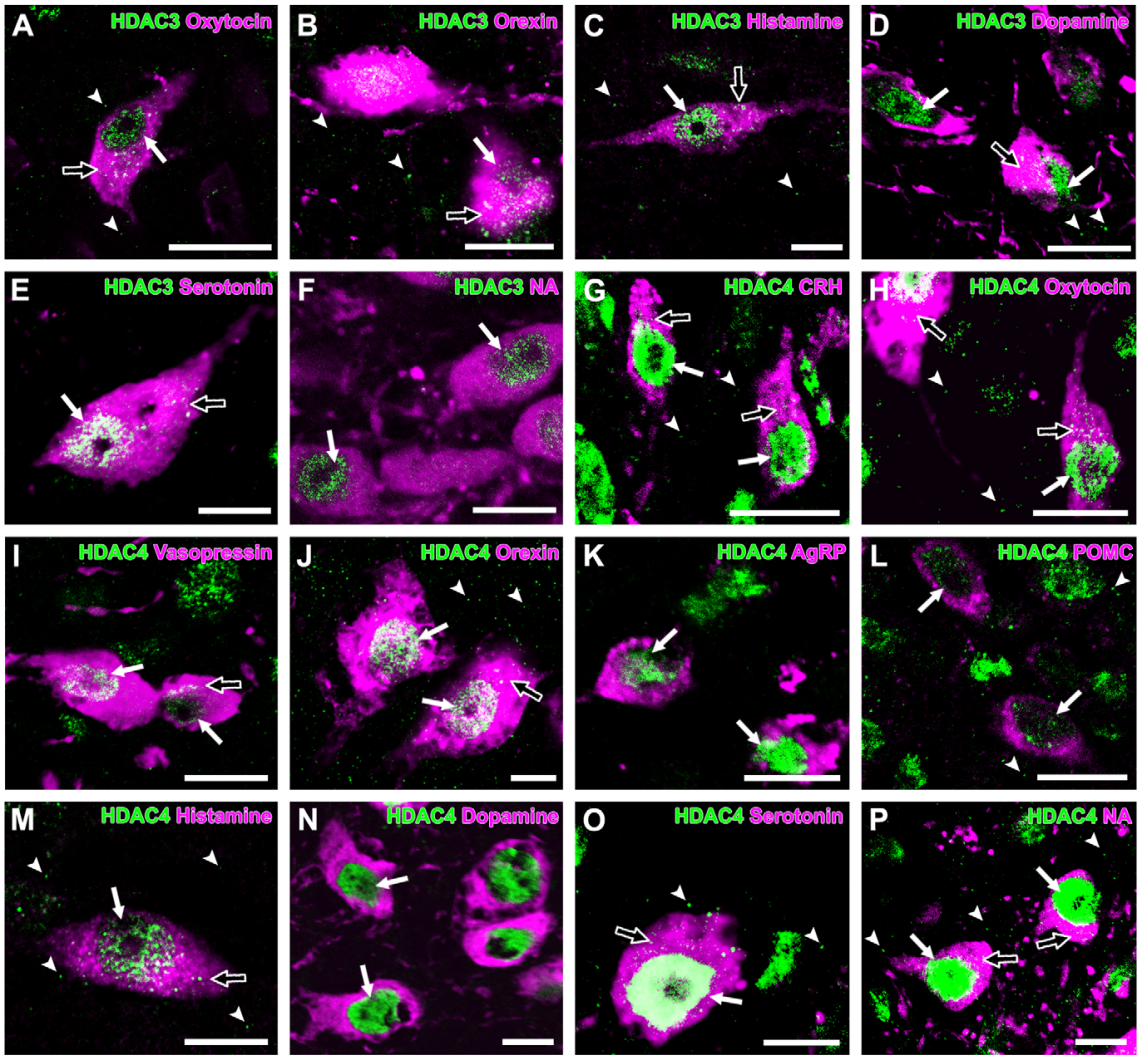
Expression of HDAC3 and -4 in monoaminergic and neuropeptidergic neurons. The HDAC immunolocalizations (green) were visualized with a neuronal marker (magenta). **A:** HDAC3 was detected in the nucleus (arrow) and cytoplasm (open arrow) of oxytocin neuron, with punctate immunoreactivity (arrowheads) in the PVN. **B:** HDAC3 was detected in the nucleus (arrow) and cytoplasm (open arrow) of orexin neurons, with punctate immunoreactivity (arrowheads) in the LHA. **C:** HDAC3 was detected in the nucleus (arrow) and cytoplasm (open arrow) of histamine neuron, with punctate immunoreactivity (arrowheads) in the TMN. **D:** HDAC3 was detected in the nuclei (arrows) and cytoplasm (open arrow) of dopamine neurons, with punctate immunoreactivity (arrowheads) in the VTA. **E:** HDAC3 was detected in the nucleus (arrow) and cytoplasm (open arrow) of serotonin neuron. **F:** HDAC3 was detected in the nuclei (arrows) of noradrenaline neurons. **G**: HDAC4 was localized in the nuclei (arrows) and cytoplasm (open arrows) of CRH neurons, with punctate immunoreactivity (arrowheads) in the PVN. **H:** HDAC4 was detected in the nucleus (arrow) and cytoplasm (open arrows) of oxytocin neurons, with punctate immunoreactivity (arrowheads) in the PVN. **I:** HDAC4 was detected in the nuclei (arrows) and cytoplasm (open arrow) of vasopressin neurons. **J:** HDAC4 was detected in the nuclei (arrows) and cytoplasm (open arrow) of orexin neurons, with punctate immunoreactivity (arrowheads) in the LHA. **K:** HDAC4 was detected in the nuclei (arrows) of AgRP neurons. **L:** HDAC4 was detected in the nuclei (arrows) of POMC neurons, with punctate immunoreactivity (arrowheads) in the ARC. **M:** HDAC4 was detected in the nucleus (arrow) and cytoplasm (open arrow) of histamine neuron, with punctate immunoreactivity (arrowheads) in the TMN. **N:** HDAC4 was detected in the nuclei (arrows) of dopamine neurons. **O:** HDAC4 was detected in the nucleus (arrow) and cytoplasm (open arrow) of serotonin neuron, with punctate immunoreactivity (arrowheads) in the DR. **P:** HDAC4 was detected in the nuclei (arrows) and cytoplasm (open arrows) of noradrenaline neurons, with punctate immunoreactivity (arrowheads) in the LC. Scale bars indicate 10 µm.

In general, HDAC3 immunoreactivity was observed in the nuclei of neurons throughout the brain. HDAC3-immunoreactive puncta were observed in the cytoplasm and neuropils. HDAC3 immunoreactivity was found in both the nuclei and cytoplasm of neuron groups, including the CRH neurons (100±0%), the oxytocin neurons (95±5%), the vasopressin neurons (100±0%), the orexin neurons (100±0%), the AgRP neurons (100±0%), the histamine neurons (92±5%), the dopamine neurons (100±0%), the serotonin neurons (100±0%), and the noradrenaline neurons (100±0%). However, a small population of POMC neurons (21±11%) did not show any immunoreactivity for HDAC3 (Figures 2A–F, Table 2). HDAC3-immunoreactive puncta in the neuropils were uniform in size and widely distributed in the PVN, LHA, ARC, TMN, and VTA. Similar to HDAC1, a minor population of HDAC3-immunoreactive puncta in the neuropils was found in dendrites. Only a few HDAC3-immunoreactive puncta in the neuropils were colocalized with PSD95-immunoreactive puncta.

### HDAC4

In the hypothalamus, HDAC4 immunoreactivity was found mainly in the nuclei of neurons [29]. HDAC4 immunoreactivity was recognized in the CRH neurons (100±0%), the oxytocin neurons (98±2%), the vasopressin neurons (100±0%), orexin neurons (100±0%), the AgRP neurons (100±0%), the POMC neurons (92±4%), the histamine neurons (85±7%), the dopamine neurons (100±0%), the serotonin neurons (93±2%), and the noradrenaline neurons (100±0%) (Figure 2G–P, Table 2). Confocal laser scanning microscopy showed HDAC4-immunoreactive puncta in the cytoplasm of neurons containing CRH, oxytocin, vasopressin, orexin, histamine, serotonin, and noradrenaline. AgRP and POMC neurons did not show cytoplasmic immunoreactivities for HDAC4 as previously reported [29]. The strong nuclear immunoreactivity for dopamine neurons (100±0%) in the VTA and substantia nigra was consistent with the previous report [26]. A small population of histamine neurons (15±7%) did not show any HDAC4 immunoreactivity. As previously reported [26], HDAC4-immunoreactive puncta were uniform in size and were widely distributed in the neuropil of brain areas, including the PVN, LHA, ARC, TMN, DR, and LC, and a number of HDAC4-immunoreactive puncta were colocalized with PSD95-immunoreactive puncta.

### HDAC5

**Figure 3:**
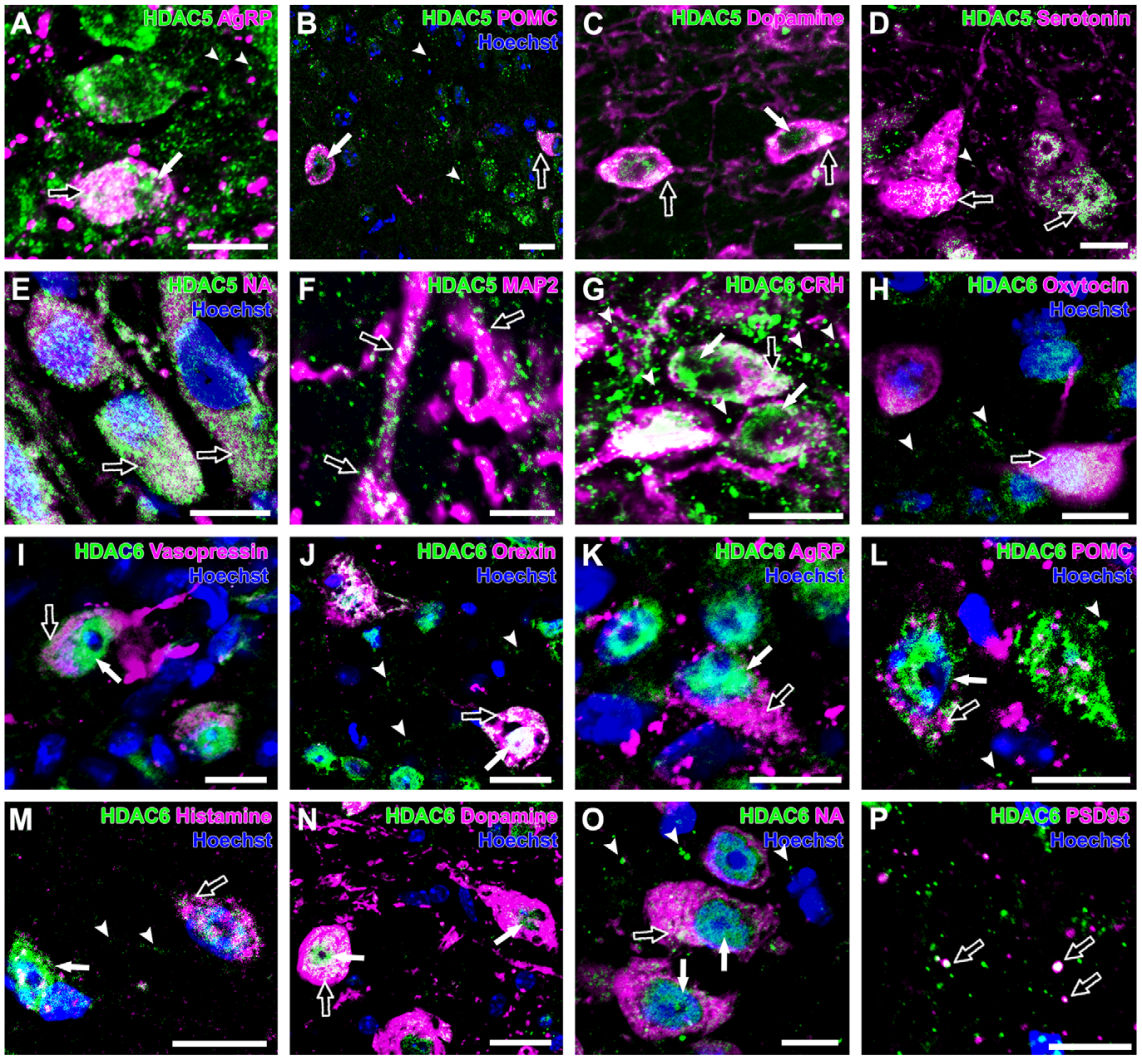
Expression of HDAC5 and -6 in monoaminergic and neuropeptidergic neurons. The HDAC immunolocalizations (green) were visualized with a neuronal marker (magenta) and Hoechst stain (blue). **A:** HDAC5 was detected in the nucleus (arrow) and cytoplasm (open arrow) of AgRP neurons, with punctate immunoreactivity (arrowheads) in the ARC. **B:** HDAC5 was detected in the nucleus (arrow) and cytoplasm (open arrow) of POMC neurons, with punctate immunoreactivity (arrowheads) in the ARC. **C:** HDAC5 was detected in the nucleus (arrow) and cytoplasm (open arrows) of dopamine neurons. **D:** HDAC5 was detected in the cytoplasm (open arrows) of serotonin neurons, with punctate immunoreactivity (arrowhead) in the DR. **E**: Strong HDAC5 immunoreactivity was observed in the cytoplasm (open arrows) of noradrenaline neurons. **F**: Granular HDAC5 immunoreactivities were observed in the dendrites (open arrows) in the LHA. **G**: HDAC6 was detected in the nuclei (arrows) and cytoplasm (open arrows) of CRH neurons, with abundant HDAC6-immunoreactive puncta (arrowheads) in the PVN. **H**: HDAC6 was detected in the cytoplasm (open arrow) of oxytocin neurons, with punctate immunoreactivity (arrowheads) in the PVN. **I**: HDAC6 was detected in the nucleus (arrow) and cytoplasm (open arrow) of vasopressin neuron. **J:** HDAC6 was detected in the nucleus (arrow) and cytoplasm (open arrow) of orexin neurons, with punctate immunoreactivity (arrowheads) in the LHA. **K:** HDAC6 was detected in the nucleus (arrow) and cytoplasm (open arrow) of AgRP neurons. **L:** HDAC6 was detected in the nucleus (arrow) and cytoplasm (open arrow) of POMC neuron, with punctate immunoreactivity (arrowheads) in the ARC. **M:** HDAC6 was detected in the nucleus (arrow) and cytoplasm (open arrow) of histamine neurons, with punctate immunoreactivity (arrowheads) in the TMN. **N:** HDAC6 was detected in the nuclei (arrows) and cytoplasm (open arrow) of dopamine neurons. **O:** HDAC6 was localized in the nuclei (arrows) and cytoplasm (open arrow) of noradrenaline neurons, with punctate immunoreactivity (arrowheads) in the LC. **P:** Some of HDAC6-immunoreactive puncta were colocalized with PSD95-immunoreactive puncta (open arrows) in the LHA. Scale bars indicate 10 µm (A–E, G–O) and 5 µm (F, P).

HDAC5 immunoreactivity was generally observed in both the nucleus and cytoplasm. HDAC5 expression was observed in the CRH neurons (100±0%), the oxytocin neurons (95±5%), the vasopressin neurons (95±3%), the orexin neurons (100±0%), the AgRP neurons (100±0%), the POMC neurons (85±1%), the histamine neurons (100±0%), the dopamine neurons (100±0%), and the serotonin neurons (100±0%)(Figures 3A–D). The noradrenaline neurons (100±0%) showed very intense immunoreactivity for HDAC5 in the cytoplasm (Figure 3E). HDAC5 was detected in the cytoplasm and dendrites of orexin neurons. Only a subset of oxytocin neurons (5±3%), vasopressin neurons (5±3%) and POMC neurons (15±1%) did not show any immunoreactivity for HDAC5 (Table 2). HDAC5-immunoreactive puncta were variable in size and distributed in the PVN, LHA, ARC, TMN, and DR (Figures 3A–E). HDAC5-immunoreactive puncta were observed in the MAP2-positive dendrites (Figure 3F). A very small population of HDAC5-immunoreactive puncta in the neuropil was overlapped with PSD95-immunoreactive puncta.

### HDAC6

HDAC6 immunoreactivity was observed mainly in the nucleus and weakly in the cytoplasm. HDAC6 immunoreactivity was found in all neurons that expressed orexin, AgRP, histamine, dopamine, serotonin, or noradrenaline. CRH neurons (95±2%), oxytocin neurons (70±7%), vasopressin neurons (80±3%), and POMC neurons (88±1%) showed variable HDAC6 immunoreactivity (Figure 3G–O, Table 2). The HDAC6-immunoreactive puncta in the cytoplasm varied in size, while the puncta in the neuropils were similarly sized and widely distributed in the PVN, LHA, ARC, TMN, VTA, DR, and LC. A small number of HDAC6-immunoreactive puncta were colocalized with PSD95-immunoreactive puncta (Figure 3P).

### HDAC7

**Figure 4:**
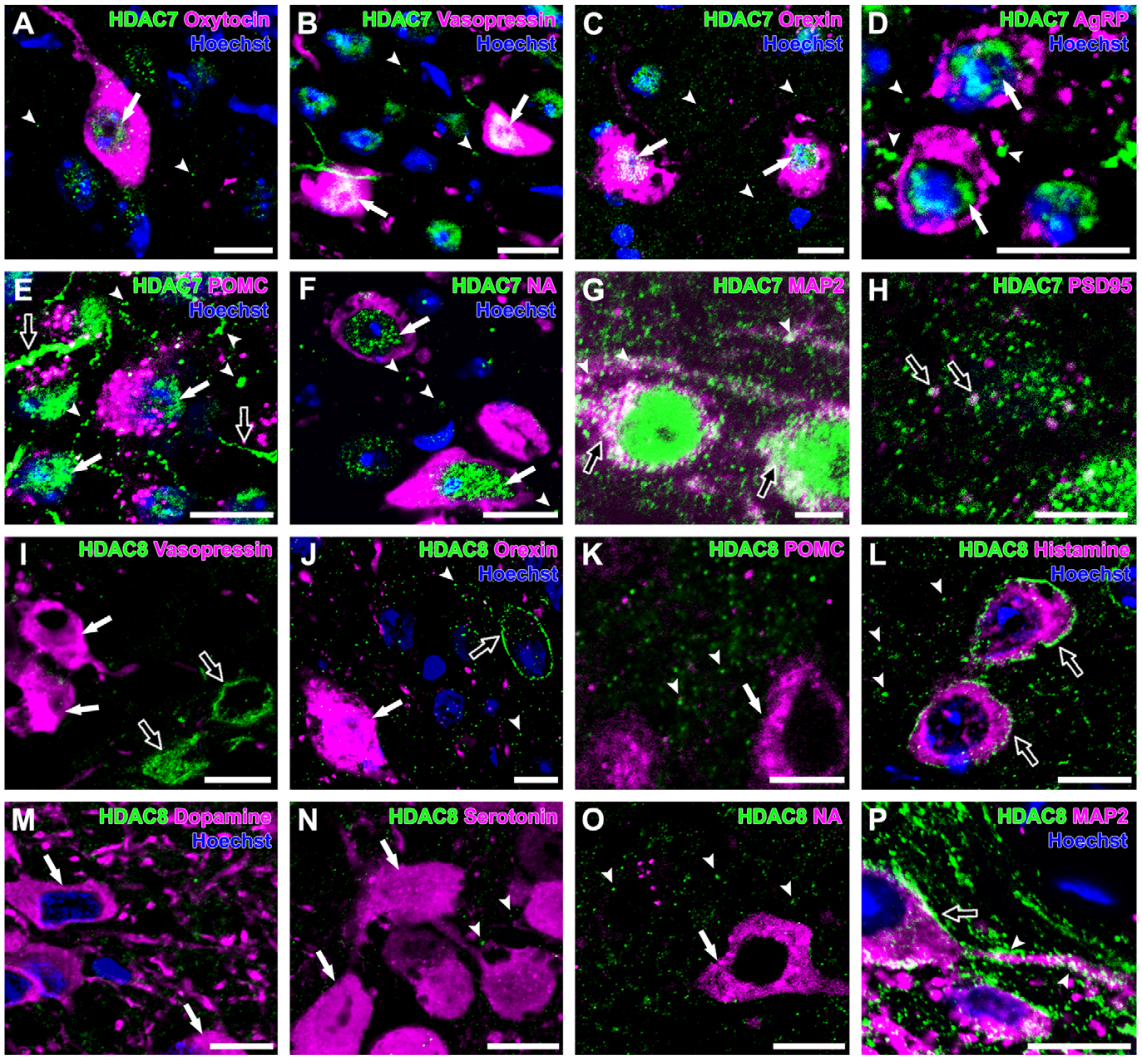
Expression of HDAC7 and -8 in monoaminergic and neuropeptidergic neurons. The HDAC immunolocalizations (green) were visualized with a neuronal marker (magenta) and Hoechst stain (blue). **A:** HDAC7 was detected in the nucleus (arrow) of oxytocin neuron, with punctate immunoreactivity (arrowheads) in the PVN. **B:** HDAC7 was detected in the nuclei (arrows) of vasopressin neurons, with punctate immunoreactivity (arrowheads) in the PVN. **C:** HDAC7 was detected in the nuclei (arrows) of orexin neurons, with punctate immunoreactivity (arrowheads) in the LHA. **D:** HDAC7 was detected in the nuclei (arrows) of AgRP neurons, with punctate immunoreactivity (arrowheads) in the ARC. **E:** HDAC7 immunoreactivity was detected in the nuclei (arrows) of POMC neuron with dendritic structures (open arrows) and puncta in neuropil (arrowheads). **F**: HDAC7 was detected in the nuclei (arrows) of noradrenaline neurons, with punctate immunoreactivity (arrowheads) in the LC. **G:** HDAC7 was detected in the cytoplasm (open arrow) and the dendrites (arrowheads) of neurons of the PVN. **H:** Some HDAC7-immunoreactive puncta were overlapped with PSD95-immunoreactive puncta (open arrows) of the LHA. **I:** HDAC8 was detected in cells (open arrows) in the PVN, but not in vasopressin neurons (arrows). **J:** HDAC8 was detected in a cell in the LHA in a pericellular pattern (open arrow), with punctate immunoreactivity (arrowheads), but not in orexin neuron (arrow). **K:** HDAC8 was not observed in POMC neuron (arrows). HDAC8-immunoreactive puncta (arrowheads) were widely distributed in the ARC. **L:** HDAC8 was detected in histamine neurons in a pericellular pattern (open arrows), with punctate immunoreactivity (arrowheads) in the TMN. **M:** HDAC8 was not observed in dopamine neurons (arrows). **N:** HDAC8 was not observed in serotonin neurons (arrows). HDAC8-positive punctate immunoreactivities (arrowheads) were widely distributed in the DR. **O:** HDAC8 was not observed in noradrenaline neuron (arrow). HDAC8-positive punctate immunoreactivities (arrowheads) were widely distributed in the LC. **P:** HDAC8 was detected in the periphery of the cytoplasm (open arrow) and dendrites (arrowheads) of the TMN neuron. Scale bars indicate 10 µm (A–F, I–P) and 5 µm (G,H).

HDAC7 immunoreactivity was found mainly in the nuclei of neurons. HDAC7 was recognized in the CRH neurons (97±2%), the oxytocin neurons (93±3%), the vasopressin neurons (87±7%), the orexin neurons (100±0%), the AgRP neurons (100±0%), the POMC neurons (99±1%), the histamine neurons (100±0%), the dopamine neurons (100±0%), the serotonin neurons (100±0%), and the noradrenaline neurons (100±0%) (Figures 4A–F). A small population of CRH neurons (3±2%), oxytocin neurons (7±3%), and vasopressin neurons (13±7%) did not show HDAC7 immunoreactivity (Table 2). HDAC7-immunoreactive puncta in the neuropil were observed in the PVN, LHA, ARC, TMN, VTA, DR, and LC (Figures 4A–F). Double immunofluorescent observation for MAP2 and HDAC7 showed that HDAC7-immunoreactivity was rich in cytoplasm and dendrites of neurons of the PVN (Figures 4G). HDAC7-immunoreactive puncta were frequently colocalized with PSD95-immunoreactive puncta (Figure 4H).

### HDAC8

HDAC8 immunoreactivity showed a clear difference among neuronal groups (Figures 4I–O). Surprisingly, all histamine neurons (100±0%) of the TMN showed HDAC8 immunoreactivity around and adjacent to histamine-immunoreactive cytoplasm, suggesting that HDAC8 was localized adjacent to the plasma membrane (Figure 4L). In addition, a small population of oxytocin neurons (4±3%) showed HDAC8 immunoreactivity in a pericellular pattern, similar to the pattern observed in the histamine neurons. In contrast, HDAC8 immunoreactivity was not recognized in any of the CRH neurons, vasopressin neurons, orexin neurons, AgRP neurons, POMC neurons, dopamine neurons, serotonin neurons, or noradrenaline neurons (Figures 4I–K, M–O, Table 2). Importantly, the majority of HDAC8-positive neurons of the brain, including the PVN, ventromedial hypothalamus, and LHA, showed HDAC8 immunoreactivity throughout the cytoplasm without a pericellular staining pattern, as previously reported (Figure 4I) [29]. HDAC8-immunoreactive puncta were uniform in size and widely distributed in the PVN, LHA, ARC, TMN, VTA, DR, and LC. The pericellular immunoreactivity was also observed along dendrites (Figure 4P). A very small population of HDAC8-immunoreactive puncta in the neuropil was overlapped with PSD95-immunoreactive puncta.

### HDAC9

**Figure 5:**
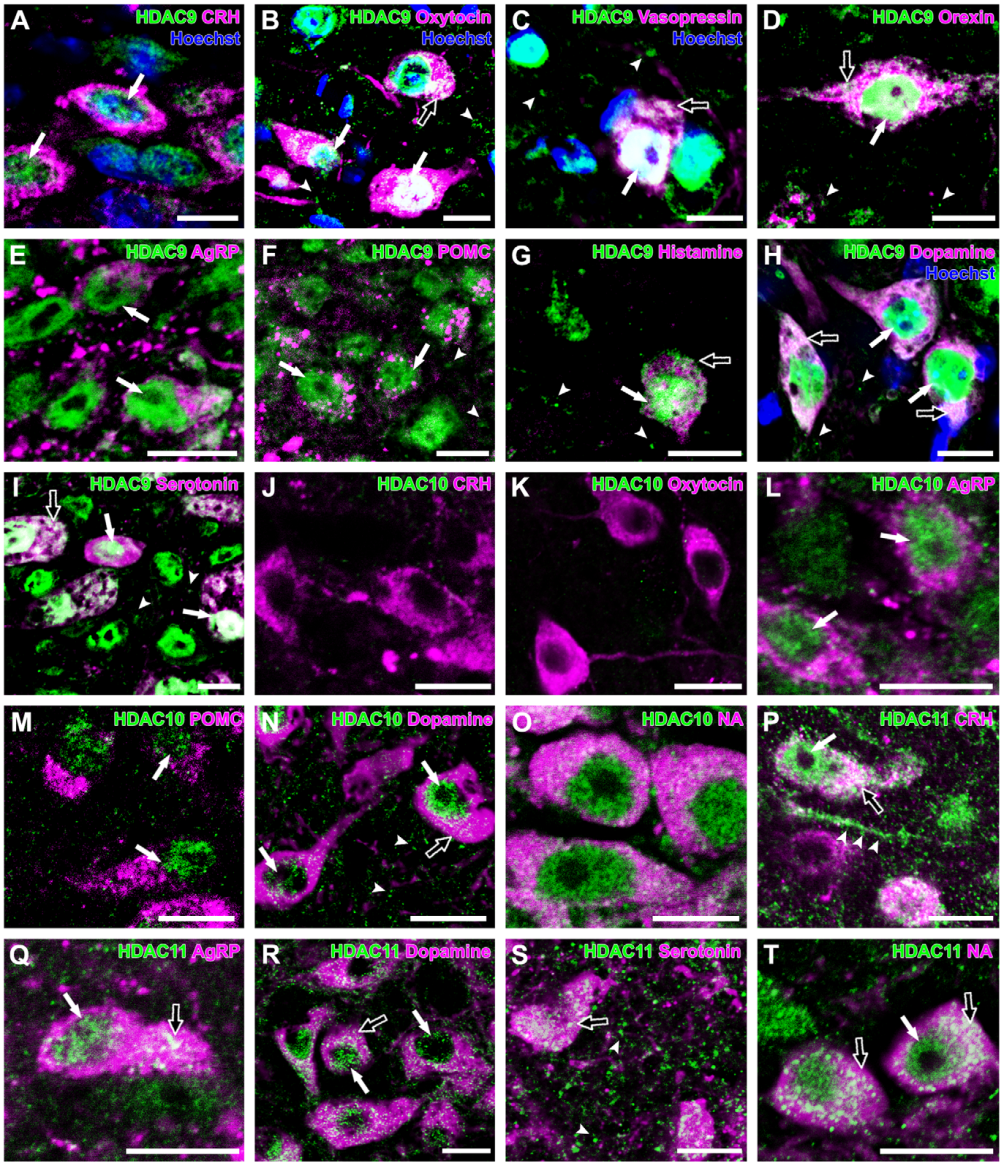
Expression of HDAC9,-10 and -11 in monoaminergic and neuropeptidergic neurons. The HDAC immunolocalizations (green) were visualized with a neuronal marker (magenta) and Hoechst stain (blue). **A**: HDAC9 was detected in the nuclei (arrows) of CRH neurons. **B**: HDAC9 was detected in the nuclei (arrows) and cytoplasm (open arrow) of oxytocin neurons, with punctate immunoreactivity (arrowheads) in the PVN. **C**: HDAC9 was detected in the nucleus (arrow) and cytoplasm (open arrow) of vasopressin neuron, with punctate immunoreactivity (arrowheads) in the PVN. **D:** HDAC9 was detected in the nucleus (arrow) and cytoplasm (open arrow) of orexin neuron, with punctate immunoreactivity (arrowheads) in the LHA. **E:** HDAC9 was detected in the nuclei (arrows) of AgRP neurons. **F:** HDAC9 was detected in the nuclei (arrows) of POMC neurons, with punctate immunoreactivity (arrowheads) in the ARC. **G:** HDAC9 was detected in the nucleus (arrow) and cytoplasm (open arrow) of histamine neuron, with punctate immunoreactivity (arrowheads) in the TMN. **H:** HDAC9 was detected in the nuclei (arrows) and cytoplasm (open arrows) of dopamine neurons, with punctate immunoreactivity (arrowheads) in the VTA. **I:** HDAC9 was detected in the nuclei (arrows) and cytoplasm (open arrow) of serotonin neurons, with punctate immunoreactivity (arrowheads) in the DR. **J:** HDAC10 was not detected in the CRH neurons. **K:** Oxytocin neurons did not show any immunoreactivity for HDAC10. **L:** HDAC10 was detected in the nuclei (arrows) of AgRP neurons. **M:** HDAC10 was detected in the nuclei (arrows) of POMC neurons. **N:** HDAC10 was detected in the nuclei (arrows) and cytoplasm (open arrow) of dopamine neurons, with punctate immunoreactivity (arrowheads) in the VTA. **O:** HDAC10 was detected in the nuclei and cytoplasm of noradrenaline neurons. **P:** HDAC11 was detected in the nucleus (arrow) and cytoplasm (open arrow) of CRH neuron and also detected in the neuropils and dendritic structures (arrowheads) in the PVN. **Q:** HDAC11 was detected in the nucleus (arrow) and cytoplasm (open arrow) of AgRP neuron. **R:** HDAC11 was detected in the nuclei (arrows) and cytoplasm (open arrow) of dopamine neurons. **S:** HDAC11 was detected in the cytoplasm (open arrow) of serotonin neurons, with punctate immunoreactivity (arrowheads) in the DR. **T:** HDAC11 was detected in the nucleus (arrow) and cytoplasm (open arrows) of noradrenaline neurons. Scale bars indicate 10 µm.

HDAC9 immunoreactivity was mainly observed in the nucleus and weakly in the cytoplasm. HDAC9 immunoreactivity was found in almost all neurons that expressed CRH (100±0%), oxytocin (100±0%), vasopressin (100±0%), orexin (100±0%), AgRP (100±0%), POMC (96±4%), histamine (96±2%), dopamine (100±0%), serotonin (100±0%), or noradrenaline (100±0%), except for a very small subset of POMC neurons (4±4%), histamine neurons (4±2%), and dopamine neurons (5±1%)(Figures 5A–I, Table 2). HDAC9-immunoreactive puncta in the cytoplasm and the neuropils were not uniform in size and were observed in the PVN, LHA, ARC, TMN, DR, VTA, and LC. A small population of HDAC9-immunoreactive puncta in the neuropil was overlapped with PSD95-immunoreactive puncta.

### HDAC10

HDAC10 showed differential expression patterns among the monoaminergic and neuropeptidergic neuron groups. HDAC10 immunoreactivity was observed in almost all neurons that expressed AgRP (98±2%), dopamine (95±1%), or noradrenaline (100±0%), whereas no HDAC10 immunoreactivity were found in CRH neurons, oxytocin neurons, vasopressin neurons, orexin neurons, histamine neurons, and serotonin neurons (Figures 5J–O). Half of POMC neuron population (55±4%) was immunoreactive for HDAC10. Whereas HDAC10 immunoreactivity within the AgRP neurons and POMC neurons was confined to the nuclei, the HDAC10 immunoreactivity of dopamine neurons and noradrenaline neurons was observed in both the nucleus and cytoplasm (Figures 5N, O, Table 2). HDAC10-immunoreactive puncta in the neuropil were observed in the PVN, LHA, ARC, TMN, DR, and LC. A small population of HDAC10-immunoreactive puncta in the neuropils was overlapped with PSD95-immunoreactive puncta.

### HDAC11

HDAC11 immunoreactivity was observed in both the nucleus and cytoplasm of almost all neurons that expressed oxytocin (89±8%), vasopressin (100±0%), orexin (100±0%), AgRP (100±0%), POMC (85±5%), histamine (100±0%), dopamine (100±0%), serotonin (100±0%), or noradrenaline (100±0%)(Figures 5P–T, Table 2). Half of CRH neurons (45±10%) were immunoreactive for HDAC11. HDAC11 immunoreactivity was also found in dendritic structures in the hypothalamus (Figure 5P). HDAC11-immunoreactive puncta were uniform in size and widely distributed in the PVN, LHA, ARC, TMN, VTA, DR, and LC, and were frequently colocalized with PSD95-immunoreactive puncta.

## Discussion

In the present study, we examined the expression profile of the HDAC protein family in monoaminergic and neuropeptidergic neurons. The expression patterns of HDAC1,-2,-3,-5,-6,-7,-9, and -11 were very similar among all monoaminergic and neuropeptidergic neurons, while the HDAC4, -8, and -10 immunoreactivity patterns were clearly different among the neuronal groups.

### Differential Expression of HDAC10 among Neuron Groups

HDAC10 expression was observed in AgRP neurons, POMC neurons, dopamine neurons and noradrenaline neurons but not in neurons containing CRH, oxytocin, vasopressin, orexin, histamine, or serotonin. Nuclear HDAC10 immunoreactivity was consistent among HDAC10-positive neurons, while cytoplasmic HDAC10 immunoreactivity was clearly observed in the dopamine neurons and noradrenaline neurons.

HDAC10, a member of the class IIb HDAC family, has a catalytic domain in the amino terminal half and is leucine-rich in the carboxyl terminal half. Although the function of HDAC10 remains largely unknown, HDAC10 is associated with hsc70, Pax3, and KAP1 and interacts with histones to enhance the deacetylated status of target molecules [34]. Another member of the class IIb HDAC family, HDAC6, has a highly similar catalytic domain as HDAC10 and deacetylates histones and cytoplasmic proteins such as alpha-tubulin, actin-binding protein, contactin, and heat shock chaperone protein HSP90 [20], [35]. Thus, HDAC10 could alter the acetylation status of a variety of nuclear and cytoplasmic molecules of neurons, resulting in a change in gene transcription and cellular function. The clear difference in HDAC10 expression among neuronal groups suggests that HDAC10 regulates gene expression levels, which are pivotal for the functional and biological specificity of AgRP, POMC, dopamine, and noradrenaline neurons.

### HDAC8 Expression in Histamine Neurons

Although HDAC8 was not expressed in the neuropeptidergic and monoaminergic neurons we examined in the current study, the histamine neuron is a unique exception. HDAC8 immunoreactivity was found in the cytoplasm of all histamine neurons with a pericellular pattern. Surprisingly, the HDAC8 immunoreactivity within the histamine neurons was confined to the cytoplasmic periphery, sparing a cytoplasmic region positive for histamine. We observed that the subcellular immunoreactive pattern for HDAC8 in neurons of the mouse brain displays nucleocytoplasmic, cytoplasmic, and pericellular distribution patterns. In the amygdala, cerebral cortex, hippocampus, and hypothalamus, a small population of neurons showed moderate to strong HDAC8 immunoreactivity in the cytoplasm and dendrites [29]. Confocal observation of HDAC8 immunoreactivity also identified cytoplasmic expression of HDAC8 in the amygdala, hippocampus, and cerebral cortex (data not shown).

HDAC8 belongs to the class I HDAC family; can deacetylase all core histones; and is associated with EST1B, Hsp70, Hsp90 and STIP [20], [36]. In smooth muscle cells, HDAC8 is co-localized with alpha-smooth muscle actin filament [37] and is interacts directly with it [38]. Although the function of HDAC8 in neurons remains unknown, the abundant HDAC8 localization in the peripheral region of the cytoplasm and dendrites of a specific subset of neurons, including histamine neurons, suggests that an undiscovered role of HDACs is in intracellular signaling rather than gene transcription. Although we previously found that a subset of neurons in the anterior parvicellular and periventricular subdivisions of the PVN changed HDAC8 immunoreactivity in response to fasting and high-fat diet feeding [29], none of the PVN neurons that expressed CRH, oxytocin, or vasopressin were positive for HDAC8. This result is consistent with the localization of only a few neurons containing CRH, oxytocin, or vasopressin in the anterior parvicellular and periventricular subdivisions of the PVN [39].

### Differential Expression Profiles among Neuron Groups

All groups of monoaminergic and neuropeptidergic neurons showed immunoreactivity for HDAC4, but the subcellular distribution and intensity varied. Cytoplasmic immunoreactivity for HDAC4 was not observed in AgRP neurons, POMC neurons, or dopamine neurons; however, cytoplasmic HDAC4 was detected in other neuronal groups. Interestingly, almost all neurons showing cytoplasmic HDAC4 immunoreactivity did not have immunoreactivity for HDAC10 (Table 2). The only exception was in the noradrenaline neurons, which showed both HDAC10 immunoreactivity and cytoplasmic HDAC4 immunoreactivity.

**Figure 6:**
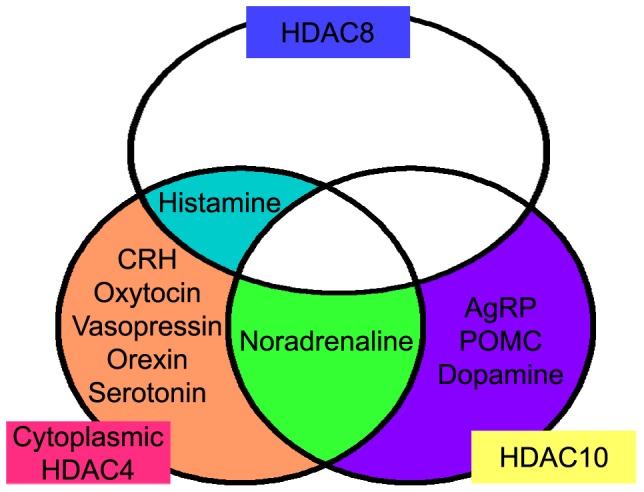
Differential HDAC expression within the monoaminergic and neuropeptidergic neurons of adult male mice.

Thus, based on the HDAC4, -8, and -10 immunoreactivities, the monoaminergic and neuropeptidergic neurons we examined were classified into four groups: 1) HDAC8-positive: histamine neurons; 2) HDAC10-positive and cytoplasmic HDAC4-negative: AgRP neurons, POMC neurons and dopamine neurons; 3) HDAC10-positive and cytoplasmic HDAC4-positive: noradrenaline neurons; and 4) HDAC10-negative and cytoplasmic HDAC4-positive: CRH neurons, oxytocin neurons, vasopressin neurons, and serotonin neurons (Figure 6). Although this classification is valid for the adult male mouse under basal conditions, it could differ based on gender, age, stress, and nutrition. Importantly, HDAC4 showed activity-dependent translocation from the nucleus to the cytoplasm *in vitro* [25], suggesting that the subcellular localization of HDAC4 could be dynamically regulated in response to environmental stimuli. Generally, most monoaminergic and neuropeptidergic neurons express HDACs in an all-or-none manner, but POMC neurons showed a variable population of HDAC-positive cells, especially for HDAC3 and HDAC10. This variable expression may be associated with the differential expression of the anorexigenic genes of POMC neurons, by modulating the acetylation status of genes important for feeding and body weight regulation when a mouse is fasted or fed a high-fat diet.

### HDACs Immunoreactivity in the Neuropils

We observed HDAC-immunoreactive puncta in the neuropil. The number of puncta varied among both HDACs and brain regions. Double immunofluorescent observation of HDACs with MAP2 showed that HDACs-immunoreactive puncta were frequently localized in the dendrites. Puncta immunoreactive for HDAC4 and -11 showed frequent colocalization with PSD95-immunoreactive puncta [26], but the majority of PSD95-immunoreactive puncta were negative for HDACs. Although the substrates and functions of HDACs in the dendrites or spines are unknown, punctate distribution suggests that the HDACs exit in a functional compartment which may be involved in molecular traffic between the cell body and spines, or spine activity [40], [41].
